# Clozapine-Induced Microseizures, Orofacial Dyskinesia, and Speech Dysfluency in an Adolescent with Treatment Resistant Early Onset Schizophrenia on Concurrent Lithium Therapy

**DOI:** 10.1155/2017/7359095

**Published:** 2017-08-01

**Authors:** Vivekananda Rachamallu, Ayman Haq, Michael M. Song, Manish Aligeti

**Affiliations:** ^1^Texas Tech University Health Sciences Center School of Medicine, Department of Psychiatry, Lubbock, TX, USA; ^2^Texas Tech University Health Sciences Center School of Medicine, Lubbock, TX, USA; ^3^Texas Tech University Health Sciences Center School of Medicine and Graduate School of Biomedical Sciences, MD/PhD Program, Lubbock, TX, USA

## Abstract

Clozapine is an atypical antipsychotic used in the treatment of refractory schizophrenia. It has a well-known side effect profile, including agranulocytosis, decreased seizure threshold, and tardive dyskinesia. In addition, numerous case reports have described clozapine-induced stuttering in adults. However, there has been only one previous case report describing it in the adolescent population. In addition, concurrent lithium therapy has been shown to enhance the neurotoxic effects of antipsychotics and lower the seizure threshold. Here, we report on the development of clozapine-induced microseizures, orofacial dyskinesia, and stuttering in a 17-year-old adolescent male with treatment of refractory early onset schizophrenia on clozapine and concurrent lithium therapy. The patient's symptoms of schizophrenia responded well to the clozapine regimen. However, with the escalating dose of clozapine, the patient developed speech dysfluency in the form of stuttering and perioral twitching. An electroencephalogram confirmed seizure activity. Due to similarities with tardive dyskinesia, symptoms of microseizures induced by atypical antipsychotics may not be accurately diagnosed. A multidisciplinary treatment of speech dysfluency is of particular importance in the adolescent schizophrenic patients, who are expected to have longer duration of lifetime exposure to antipsychotics and in whom peer group interaction is crucial for normal personal and social development.

## 1. Introduction

Clozapine is a second-generation antipsychotic that is particularly useful in the management of treatment resistant schizophrenia [1, 2]. Some of the commonly observed side effects of clozapine include agranulocytosis, decreased seizure threshold, weight gain, cardiomyopathy, orthostatic hypotension, sialorrhea, sedation, constipation, hyperglycemia, and dyslipidemia, while extrapyramidal symptoms such as tardive dyskinesia are relatively less frequently observed [2, 3]. In a case series reporting 6 cases of clozapine-induced stuttering, Murphy et al. reported an incidence of 0.92% of clozapine-induced stuttering among 654 Irish patients receiving clozapine [4]. In a 2012 case report on clozapine-induced stuttering, Grover et al. reported 16 previous case reports on clozapine associated stuttering [5]. Clozapine-induced stuttering has been reported to be dose dependent, with increased risk with dose escalation [6, 7]. Although clozapine has been reported to demonstrate higher incidence of adverse drug events in the pediatric population [8], to date, there is only one case report describing clozapine-induced stuttering in the adolescent population [9]. As for the combination treatment regimens, the combination of antipsychotics with lithium has been in clinical use for many decades to enhance treatment response [10]. Here, we report on the development of clozapine-induced microseizures with perioral twitching and stuttering in a 17-year-old adolescent male with treatment refractory early onset schizophrenia who was started on clozapine. The patient was on concurrent lithium therapy at the time of the start of clozapine treatment. An electroencephalogram was performed which confirmed seizure activity. In early onset schizophrenia, patients will likely be exposed to a higher lifetime doses of antipsychotic medications, increasing their potential to developing even the rarest of side effects. In addition, the potential adverse effect of antipsychotic-induced speech dysfluency on personal and social development necessitates a multidisciplinary care with a careful consideration of all possible treatment and support modalities.

## 2. Case Description

The patient is a 16-year-old adolescent Caucasian male with a past history of diabetes mellitus type 1 (diagnosed at age 10) with history of diabetic ketoacidosis (DKA) with episodic hallucinations, gastroesophageal reflux disease (diagnosed at age 10, s/p fundoplication), cerebral contusion (at age 14) without any history of seizures, and history of occasional cocaine use. At the time of initial psychiatric evaluation, the patient was admitted for DKA with persisting visual hallucinations lasting for more than 48 hours, even after adequate glucose control. His symptoms included hallucinations of hearing screams and voices telling him to harm himself and others. A more detailed description of the patient's symptoms can be found in Table 1. The patient's family history included schizophrenia in paternal grandfather and two paternal aunts, substance use disorders in paternal grandfather, depression in mother, and stuttering in father which spontaneously resolved during his adolescence. Initial laboratory workup of the patient revealed no abnormalities other than poor glycemic control. A baseline MRI of the brain showed no acute or chronic intracranial pathology. Thyroid disease and other neurological and systemic conditions that may cause psychosis were ruled out and urine drug screen was also negative. The patient's psychotic symptoms resolved completely with a trial of haloperidol and the patient was discharged with a psychiatry clinic follow-up visit.

Following the initial evaluation, the patient received psychiatric care on an outpatient basis for the ensuing couple of years. The patient's psychotic symptoms continued to worsen; he reported thought broadcasting on television and paranoia about hidden microphones in the walls at home which were there to spy on his thoughts. The patient's visual hallucinations also continued to worsen and he reported seeing his leg being cut off, seeing his brother being stabbed, and becoming violent and homicidal, which resulted in 2 additional psychiatric hospitalizations. He also became withdrawn and isolated, as well as not being able to attend or perform school work. He also developed depressive mood symptoms which ultimately resulted in the diagnosis of schizoaffective disorder. For a detailed timeline of the patient's clinical progression of his psychotic symptoms, see Table 1. During those years, until the start of clozapine therapy, the patient's psychiatric medication history included aripiprazole, risperidone, olanzapine, haloperidol, divalproex sodium, benztropine, clonazepam, fluoxetine, and citalopram. The reasons for each change in the medication regimen included poor tolerability, worsening symptoms, or insufficient response at the maximum tolerated dose. For a detailed timeline of the patient's clinical history, see Table 1.

**Figure 1:**
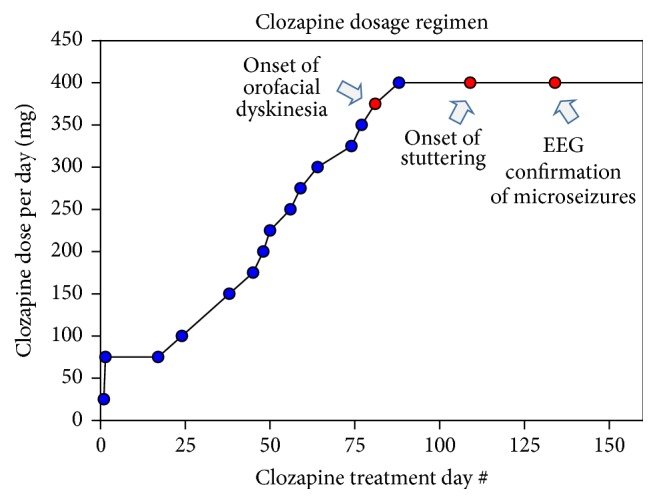
Dose escalation and key events in the course of the patient's clozapine therapy. On Day 334 of clozapine therapy: dose = 400 mg per day, level = 541 ng/ml (pharmacokinetic study results included in the clozapine package insert report peak concentrations of 102–771 ng/ml and minimum steady state concentrations of 41–343 ng/ml [11]). The patient was also on concurrent lithium 300 mg PO BID on Day 1 of clozapine therapy which was subsequently increased to 450 mg PO BID on Day 24 of clozapine therapy. The patient did not demonstrate any symptoms of lithium toxicity.

Due to the lack of improvement with other antipsychotics, the patient was started on clozapine. The patient's other medications at the time included lithium, citalopram, clonazepam, and atenolol. During the clinic visit at Day 38 of clozapine therapy, with the dose of 100 mg per day, the patient demonstrated a modest improvement in his visual and auditory hallucinations. He demonstrated no extrapyramidal symptoms and his Abnormal Involuntary Movement Scale (AIMS) score was zero. The patient's dose of clozapine was gradually escalated on an outpatient basis and the progress of the dose escalation is summarized in Figure 1. At Day 77, with the clozapine dose of 350 mg per day, the patient developed rare infrequent orofacial dyskinesia with perioral twitching. Due to continuing improvement in the patient's symptoms of schizophrenia, the dose was continued to be escalated. However, due to the anxiety regarding potential worsening of the side effects, the patient and the parents requested that the dose escalation be carried out at a slower pace. Then at Day 109, with the clozapine dose of 400 mg per day, the patient developed persistent stuttering. The patient and the mother reported previous anxiety-related intermittent stuttering that occurred approximately once every several months, each with duration of a few minutes. However, his stuttering worsened significantly with clozapine 400 mg per day and the stuttering was now constant and no longer related to his mood. The patient had an especially hard time with pronunciation of letters “I,” “D,” and “T” and was minimizing verbal communication while in public places and increasingly relying on his parents to facilitate his communication. However, the patient demonstrated significant improvement in both positive and negative symptoms, no longer had suicidal ideations, and was socially engaged and able to continue his school work.

**Figure 2:**
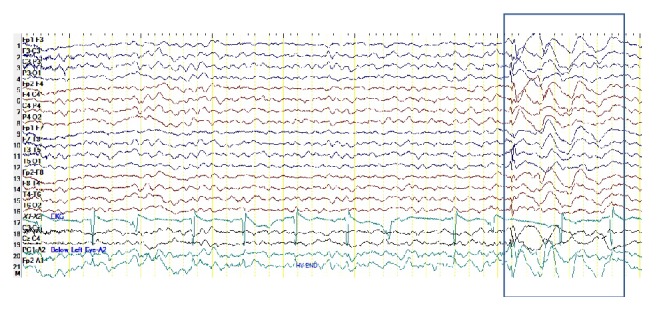
Electroencephalogram (EEG) demonstrating microseizures. The EEG was obtained on Day 134 of clozapine therapy [Day 88: clozapine dose escalation to 400 mg per day (with concurrent lithium 450 mg PO BID), Day 109: development of stutter].

At Day 134, with continuation of mild intermittent perioral twitching and persistent stuttering, an electroencephalogram was performed. The study showed an abnormal waking and sleep EEG (Figure 2). Several episodes of generalized spike and wave activity of 3 Hz that lasted 2-3 seconds during photic stimulation and hyperventilation suggested epileptiform activity. Due to the intermittent form of this activity, we will hereafter refer to it as a “microseizure.” In spite of the worsened stutter, clozapine was continued at the same dose as psychotic symptoms and suicidal thoughts were finally well controlled and the patient's functionality returned close to his baseline. Due to the possibility of potentiation of clozapine-induced side effect by lithium, a change of lithium to divalproex sodium was recommended. However, the patient and the parents refused the switch as the patient had not had such an excellent response to treatment since his initial diagnosis. The patient was referred to a speech language pathologist for evaluation, therapy, and support. In subsequent clinic visits, due to the worsening symptoms of stuttering, the patient and the parents decided to proceed with the tapering-off of lithium and the start of divalproex sodium. After 4 weeks of divalproex sodium 500 mg BID, the patient and the family reported improvement in perioral twitching and stuttering. The patient is currently on clozapine and divalproex sodium combination therapy and continues to receive coordinated outpatient based care from a pediatrician, child and adolescent psychiatrist, and neurologist and speech language pathologist. The patient continues to require close monitoring including the management of positive and negative symptoms of schizophrenia, as well as ongoing monitoring for any development of generalized tonic-clonic seizures. The patient has not had any further psychiatric hospitalizations since starting clozapine therapy.

## 3. Discussion

Early onset schizophrenia has a prevalence of 1-2 per 1000 and is associated with severe brain abnormalities, poor psychosocial functioning, and overall poorer clinical outcomes [12]. To complicate the picture, adolescents who suffer from this are more likely to face social rejection, have poor peer relationships, and have more academic trouble compared to adult onset schizophrenia [12]. Clozapine is effective in treatment resistant schizophrenia, but some of its side effects such as stuttering may worsen the social dysfunction in the adolescent patient population.

Stuttering itself is a very complex phenomenon that does not have a unifying pathophysiology, despite some common neurological changes in those who stutter [13]. It is defined as an involuntary disturbance in normal fluency or speech pattern and is characterized by sound repetitions, sound prolongations, or broken words that lead to marked speech dysfluency [7]. Stuttering has been reported to be one of the side effects resulting from the first- and second-generation antipsychotics, including clozapine [14–17]. Furthermore, clozapine-induced stuttering has been associated with microseizure-like activity confirmed with EEG [5, 9]. In a case report by Horga et al., a patient developed speech disfluency with myoclonus with clozapine 350 mg per day and clomipramine 225 mg per day and subsequently had generalized tonic-clonic seizures [9]. In another case report by Duggal et al., a patient developed stuttering when the dose of clozapine was increased to 300 mg per day and eventually developed tonic-clonic seizures when the clozapine dose was increased to 425 mg per day [18].

While our case also showed seizure-like activity on an EEG that corresponded to the patient's stuttering, there is a discrepancy in the current literature about the etiology of the stuttering. Murphy et al. reported that most of the clozapine-induced symptoms resolved without any antiepileptic treatment and two patients with clozapine-induced seizures showed no EEG abnormalities [4], although a lack of EEG changes does not necessarily preclude the diagnosis of seizures in a clinical setting. The role of antiepileptic pharmacotherapy in cases of clozapine-induced stuttering remains yet unclear, although antiepileptic therapies may have a role in preventing tonic-clonic seizures. It certainly would be advisable for the patient to be monitored for any emerging signs of seizures.

Clozapine-induced stuttering has been reported to be dose dependent, with increased risk with dose escalation [6, 7]. The potential to cause microseizures may be increased not only with a high dose but also with quicker titration. Murphy et al. reported that several patients developed stuttering when their dosage was titrated too rapidly or the target dose was too high and the symptoms resolved with a reduction of the dose by 25–50 mg or with a slower titration schedule [4]. However, they also reported that one of the patients developed stuttering without any changes in the medication regimen and a dose reduction resulted in no change in the side effect but a recurrence of their psychosis [4].

It is also important to note that our patient was on concurrent lithium therapy. It has been suggested that lithium enhances the neurotoxic effects of first- and second-generation antipsychotics including clozapine, in regard to seizures and extrapyramidal symptoms [10, 19, 20]. However, we are unaware of any previous reports of explicit link between lithium and atypical antipsychotic-induced stuttering.

Any role of recently approved valbenazine and its class of drugs (which were developed for the treatment of tardive dyskinesia) in the treatment of stuttering and microseizures is unknown [21]. Rather, it is important to differentiate them from extrapyramidal symptoms such as tardive dyskinesia and vocal tics, especially in adolescent patients. The recognition of stuttering and microseizures and proper multidisciplinary treatment approach are of particular importance in the adolescent schizophrenic patients in whom social interaction and peer group acceptance is crucial for normal personal and social development.

To our knowledge, this is the second reported case of clozapine associated stuttering in the adolescent population that shows epileptiform EEG activity. However, our patient had a positive family history and a previous history of emotionally induced intermittent stuttering, prior to the development of persistent stuttering with the clozapine plus lithium regimen. The intermittent occurrence of microseizures and orofacial dyskinesia, compared to the persistent nature of the stuttering and speech dysfluency with clozapine and lithium therapy, indicates that the stuttering likely results from a more complex etiology.

Due to the increased use of clozapine in pediatric population with psychosis and subsequent increase in reported incidence of adverse drug reactions [8], even the rarest of side effects of clozapine (such as microseizures and stuttering) are more likely to be observed. That would especially be the case in early onset schizophrenia patients who will likely face a higher lifetime exposure to antipsychotic pharmacotherapy. Clozapine associated speech dysfluency on personal and social development necessitates a multidisciplinary care with a careful consideration of all possible treatment and support modalities. There are studies, albeit few, that report on the efficacy of speech therapy programs utilized in the treatment of stuttering in the child and adolescent patient population [22–24]. Although the underlying etiology and pathology of our patient's stuttering may be distinct from other cases of neurogenic stuttering, some of the approaches utilized in various speech therapy programs would likely provide at the least coping mechanisms and supportive structures.

Clinicians must be more discerning in differentiating microseizures and stuttering from extrapyramidal symptoms or vocal tics. Patients who develop microseizures may need slower titration of clozapine with close monitoring for seizure activity. Treatment of microseizures with antiepileptics may also be warranted, particularly because microseizures may be a risk factor in the development of generalized tonic-clonic seizures [18].

## 4. Conclusion

Clozapine associated stuttering is a particularly significant side effect in the adolescent population because of the adverse impacts it can have on social interactions. Because the adolescent population relies heavily on peer group for social support, stuttering may lead to social withdrawal or noncompliance. Speech evaluation and supportive psychotherapy should be explored as alternatives or adjuncts to antiepileptics, as ways to reduce the social consequences of this unique side effect. There have been some reports focused on the link between the clozapine-induced stuttering and how it is related to seizure activity in EEG. However, more research focused on potential treatment and rehabilitation approaches is needed.

## Figures and Tables

**Table 1:** Summary of patient's clinical progress.

Date	Clinical event(s)	Description
May 2013	ED visit for poor glucose control and episodic visual hallucinations	The patient was diagnosed with Type 1 DM in 2009. The patient reported episodic visual hallucinations during the ED visit for diabetic ketoacidosis (DKA).

January 2015	Hospitalized for persistent visual and auditory hallucinations. Psychiatry service was consulted for psychiatric evaluation	During in-patient psychiatric evaluation, the patient endorsed visual hallucinations of seeing a tall man and little girls who he thought were stalking him. He also reported auditory hallucinations of hearing screams and voices telling him to harm himself and others. The symptoms persisted even after glucose levels were under control. The patient reported positive family history of schizophrenia in paternal grandfather and two paternal aunts. The psychotic symptoms resolved completely after several days of haloperidol while he was in the hospital.

February 2015	First psychiatry clinic visit	The patient reported having hallucinations of somebody touching his shoulder and whispering in his ears. CT and MRI were negative for acute intracranial pathology. The patient also reported sadness and was withdrawn as per family. He reported being anxious and having 7-8 panic episodes per day, often when he thinks about hallucinations. He denied current use of recreational drugs and the urine drug screen (UDS) was negative. Thyroid function was WNL. The patient was started on fluoxetine 10 mg PO daily and Cognitive Behavioral Therapy for Anxiety.

March 2015	Psychiatry clinic visit	The patient continued to experience hallucinations. He reported improvement with anxiety. He stopped taking insulin and wrote his plan for suicide. Fluoxetine increased to 20 mg PO daily and aripiprazole 5 mg PO daily was started to address psychotic symptoms and depression.

April 2015	Psychiatry clinic visit	The patient became violent and threatened family member with homicidal statements. Due to worsening symptoms, aripiprazole was switched to risperidone 1 mg PO BID. Fluoxetine continued at 20 mg PO daily.

July 2015	Psychiatry clinic visit	The patient's psychotic symptoms worsened. He reported visual hallucinations of his brother being stabbed by two men and voice telling him to kill himself. UDS screen is negative. Due to the worsening symptoms while on risperidone and family history of good response to olanzapine, the patient was switched to olanzapine 5 mg PO QHS.

October 2015	Psychiatry clinic visit	The patient reported easily getting agitated. His school grades were dropping and he demonstrated less peer interaction. The patient was switched to home schooling. Psychotic symptoms were stable for few a months but worsened for the several weeks prior to the clinic visit. He threatened his family members. Olanzapine increased from 10 mg to 15 mg PO QHS.

December 2015	In-patient psychiatric hospitalization	The patient was hospitalized for worsening psychosis and violent behavior. The patient's clinical evaluations and psychological testing confirm the diagnosis of schizoaffective disorder, bipolar type. Olanzapine was placed on hold and the patient received haloperidol for worsening psychotic symptoms during the in-patient stay. The patient's symptoms stabilized with haloperidol. The patient was discharged on haloperidol.

February 2016	Psychiatry clinic visit	The patient's psychotic symptoms worsened after discharge, even with the increase of haloperidol to 10 mg PO BID. Lithium 300 mg PO BID was added for mood stabilization.

March 2016	In-patient psychiatric hospitalization	Patient was admitted due to worsening of psychosis and family member being unable to take care of him at home. The patient reported thought broadcasting on television and paranoia about hidden microphones in the walls at home which were there to spy on his thoughts. Haloperidol was put on hold and the patient was started on clozapine. The dose of lithium was increased to 450 mg PO BID.

May 2016	Psychiatry clinic visit	The patient's clozapine dose was 350 mg per day (with lithium continuing at 450 mg PO BID). The patient reported “rare mouth movements.” The patient and family reported improvement of psychotic symptoms and aggressive behavior.

June 2016	Psychiatry clinic visit	The patient's clozapine dose was 400 mg per day (with lithium continuing at 450 mg PO BID). The patient reported worsening of stuttering. The patient and family reported continuing improvement of psychotic symptoms and aggressive behavior.

July 2016	Psychiatry clinic visit	The patient's clozapine dose was 400 mg per day (with lithium continuing at 450 mg PO BID). The patient and family reported continuing improvement of psychotic symptoms and aggressive behavior. There was further worsening of stuttering; EEG demonstrates epileptiform activity (Figure 2). Family refused to change medications as the patient has excellent response with clozapine and lithium and was near resolution of his psychotic symptoms.

February 2017	Psychiatry clinic visit	The patient's clozapine dose was increased to 450 mg per day (with lithium continuing at 450 mg PO BID) due to reported recurrence of visual hallucinations.

March 2017	Psychiatry clinic visit	The patient's clozapine dose was 450 mg per day (with lithium continuing at 450 mg PO BID). Family reported worsening of stuttering but refused any changes in doses or alterations in medication regimen due to concerns of relapse of psychotic symptoms.

May 2017	Psychiatry clinic visit	Clozapine dose was 450 mg per day. Family reported worsening of stuttering and significant impairment in social interaction due to stuttering. However, the patient's psychotic symptoms remain well under control. The patient and family agreed to switch from lithium to divalproex sodium. Divalproex sodium was started at 500 mg PO BID.

June 2017	Psychiatry clinic visit	Clozapine dose was 450 mg per day (with divalproex sodium 500 mg PO BID). Family reported improvement with stuttering. The patient is able to socialize with family and friends. The patient's psychotic symptoms remain well under control.
